# Whole Exome Sequence Analysis for Inborn Errors of IL-12/IFN-*γ* Axis in Patient with Recurrent Typhoid Fever

**DOI:** 10.1155/2023/1761283

**Published:** 2023-02-17

**Authors:** Faaiz ul Hassan, Mohammed M. Aljeldah, Fozia Fozia, Mubbashir Hussain, Taj Ali Khan, Sami Siraj, Ijaz Ahmad, Muhammad Qasim, Imran Khan, John P. Giesy, Mourad A. M. Aboul-Soud

**Affiliations:** ^1^Department of Microbiology, Kohat University of Science and Technology, KP, Kohat 26000, Pakistan; ^2^Department of Clinical Laboratory Sciences, College of Applied Medical Sciences, University of Hafr Al Batin, Hafr Al Batin 39524, Saudi Arabia; ^3^Department of Biochemistry, KMU Institute of Medical Sciences, Kohat, KP 26000, Pakistan; ^4^Institute of Pathology and Diagnostic Medicine, Khyber Medical University Peshawar, KP 25000, Pakistan; ^5^Institute of Pharmaceutical sciences, Khyber Medical University Peshawar, KP 25000, Pakistan; ^6^Department of Chemistry, Kohat University of Science and Technology, KP, Kohat 26000, Pakistan; ^7^Toxicology Centre, University of Saskatchewan, Saskatoon, SK, Canada S7N 5B3; ^8^Department of Veterinary Biomedical Sciences, University of Saskatchewan, Saskatoon, SK, Canada S7N 5B4; ^9^Department of Integrative Biology, Michigan State University, East Lansing, MI 48824, USA; ^10^Department of Environmental Sciences, Baylor University, Waco, TX 76706, USA; ^11^Chair of Medical and Molecular Genetics Research, Department of Clinical Laboratory Sciences, College of Applied Medical Sciences, King Saud University, P.O. Box 10219, Riyadh 11433, Saudi Arabia

## Abstract

**Background:**

The IL-12/IFN-*γ* axis pathways play a vital role in the control of intracellular pathogens such as *Salmonella typhi*.

**Objective:**

The study is aimed at using whole exome sequencing (WES) to screen out genetic defects in IL-12/IFN-*γ* axis in patients with recurrent typhoid fever.

**Methods:**

WES using next-generation sequencing was performed on a single patient diagnosed with recurrent typhoid fever. Following alignment and variant calling, exomes were screened for mutations in 25 genes that are involved in the IL-12/IFN-*γ* axis pathway. Each variant was assessed by using various bioinformatics mutational analysis tools such as SIFT, Polyphen2, LRT, MutationTaster, and MutationAssessor.

**Results:**

Out of 25 possible variations in the IL-12/IFN-*γ* axis genes, only 2 probable disease-causing mutations were identified. These variations were rare and include mutations in IL23R and ZNFX I. Other pathogenic mutations were found, but they were not considered likely to cause disease based on various mutation predictors.

**Conclusion:**

Applying WES to the patient with recurrent typhoid fever detects variants that are not much important as other genes in the IL-12/IFN-*γ* axis. Results of the current study suggest that a large population sizes would be needed to examine the functional relevance of IL-12/IFN-*γ* axis genes with recurrent typhoid fever.

## 1. Introduction

*Salmonella typhi* is a gram-negative rod-shaped flagellated bacteria responsible for causing typhoid fever with humans being the only reservoir host [3]. The infection caused by *S. Typhi* is predominately transmitted via a fecal-oral route, particularly by consumption of contaminated water [25]. The risk of infection is particularly great in developing countries where the infection remains endemic. Another factor that contributes to the transmission of typhoid infection includes poor sanitation and probably lack of access to safe and hygienic food and water. Enteric fever in people from developed countries is usually associated with travel to an area of high endemicity, it might also be associated with individuals preparing the food who are chronic carriers of infection [6].

In the 21^st^ century, enteric fever is responsible for causing a significant number of mortalities in high risk regions of the world [16]. The prevalence of typhoid fever is alarmingly high with approximately 200,000 deaths each year in developing countries [25]. The worldwide report indicates that approximately 27 million new typhoid cases are reported every year. The most prevalent instances with greater mortality are seen in south central and Southeast Asian countries. The study previously done indicated that among Asian countries, Pakistan ranked second in the incidence of typhoid fever with an estimated rate of 412.9 for every 100,000 individuals. Similarly, the report evaluated by The International Vaccine Institute in 2010 states that there were 11.9 million typhoid fever illnesses which resulted in 129,000 deaths in low- and middle-income nations (Yasin et al., 2018).

The course of infection of *Salmonella* begins when bacteria are ingested and settle in intestinal microfold M. cells, which is followed by the uptake of *Salmonella spp*. by macrophages and dendritic cells in the intestinal submucosa [8]. Successful elimination of *Salmonella* species depends on interactions between macrophages that have internalized *Salmonella* and T lymphocytes, despite the involvement of other cells [23]. Epithelial cells and phagocytic cells, including macrophages, neutrophils, and dendritic cells recognize the pathogen associated molecular pattern (PAMP) found in bacteria. The pattern recognition receptors (PMPs) that include NOD-like receptors (NLRs) and Toll-like receptors (TLRs) form the early arms of the innate immune system that recognize PAMPs, recruiting the signals, and activating the macrophages and neutrophils against *Salmonella* [9]. In course of primary infection caused by *Salmonella*, it has been shown that without the reflection of the *Salmonella's* function, the humoral arm of the immunity plays no significant response in killing of the bacteria [27].

Macrophages play an important role in immunity against intercellular bacteria. In addition to their roles in immunity, both macrophages and dendritic cells also secrete certain cytokines IL-12 and 1L-23, these cytokines induce IFN-*γ* production by natural killer cells and T cells, which further increases the phagolysosomal fusion, phagocytosis, oxidative burst, and other not fully elucidated, nonoxidative mechanisms [11]. The interferon (IFN-*γ*) and interleukins (IL-12, IL-18, IL-23, and IL-27) are important cytokines responsible the killing of intracellular pathogens, such as *Salmonella spp*. It has been noted that defects or changes in the production of these cytokines can cause individuals to be predisposed toward being infected with *Salmonella spp*. [23]. Mutations in any of the genes controlling any of the molecules along the IFN-*γ*/IL-12 axis could result in deficiencies in clearing infections [24] and Zhan et al., 2008). To date mutations in the seven autosomal genes, IL-12B, IL-12R*β*1, IFNGR1, IFNGR2, IRF8, STAT1, and ISG15 and X-linked genes NEMO have been described in infection caused by intracellular pathogens. All of these genes are involved in Mendelian susceptibility to mycobacterial disease [5]. Presently, 34 genetic diseases have been attributed to mutations in 18 genes responsible for causing MSMD. These mutations include IL-12B, IL-12R*β*1, IL-12R*β*2, IFNG, IFNGR1, IFNGR2, IL-23 ISG15, IRF8, ZNFX1, IKBKG, CYBB, STAT1, RORC, SPPL2, JAK1, TBX21, and TYK2 [12].

Since IL-12/IFN-*γ* plays an important role in controlling infections caused by intracellular pathogens such as *Mycobacterium tuberculosis* and *Salmonella spp*. [23], the role of IL-12/IFN-*γ* has been well documented in infection caused by *Mycobacterium tuberculosis* [11], yet it has to be explored in *S. typhi*. Since dozens of people are suffering from recurrent typhoid infection, and the importance of IL-12/IFN-*γ* in recurrent typhoid fever is not well-studied; therefore, in this study, the role and importance of the genes in the IL-12/IFN-*γ* axis via the whole exome sequence analysis of a patient with recurrent typhoid infection were analyzed.

## 2. Materials and Methods

### 2.1. Patient Clinical History and Ethical Approval

In this study, one HIV-negative, 21-year-old, female patient who was born from consanguineous parents and had a history of recurrent typhoid fever was evaluated. The relevant clinical data with blood sample was obtained from the patient. The ethical approval of the current study was obtained from ethical committee of Kohat University of Science and Technology, Kohat, Pakistan, according to the Helsinki Convention Guidelines.

### 2.2. Extraction of Genomic DNA from Blood

DNA was isolated from blood by salting out technique. This method was adopted as this procedure of DNA extraction is nontoxic and gives good quality DNA from whole blood [20].

### 2.3. DNA Quality Concentration Analysis

The concentration of the extracted DNA was analyzed through agarose gel via gel electrophoresis and nanodrop. The A260/A280 value of the extracted was also recorded.

### 2.4. Whole Exome Sequencing

Whole exome sequencing was performed on Illumina HiSeq at GENEWIZ laboratory, Suzhou Industrial Park, 215123, China. Extracted genomic DNA obtained from blood was sheared by ultrasonification. Fragmented DNA was used to construct a sequencing library, through various steps of terminal repair, including the addition of base A tail, adaptor ligation purification as well preamplification exon capture, and amplification by polymerase chain reaction (PCR). DNA was quantified by use of a Qubit Fluorometer.

### 2.5. Mapping Sequence against the Reference Genome

The DRAGEN genome pipeline was used to process sequence alignment. A bwa-mem+samtools+picard+GATK process with enhanced was used to give better efficacy of sequence alignment, sorting, marking duplication, quality correction, and detection of mutation. Besides samtool [15], in-house software, depth, coverage, mapping ratio, and duplication rate were calculated to reveal the consistency of the data and the accurateness of later calling variations.

### 2.6. Filtering and Identification of Candidate Genomic Variants in IL-12/IFN-*γ* Axis Genes

The analysis was restricted to nonsynonymous exonic variants. Variants were filtered out with a MAF>0.01 in our in-house exome database. The patient exomes were filtered for mutations in 25 genes associated with intracellular infections. The top and most likely disease causing variants were Sanger sequenced for confirmation. All the confirmed mutations were then analyzed by using various computational mutational predictors including SIFT, CADD (combined annotation –dependent depletion), Polyphen2, and MutationTaster2 [1, 13, 21, 26]; and [17].

### 2.7. Statistical Analysis

To compare between healthy and patient, a chi-square test was used. The significance level was followed as *α* = 0.05.

## 3. Results

**Figure 1:**
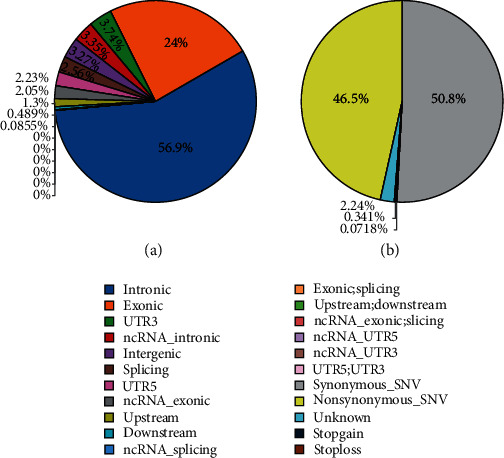
Distribution of SNVs and its effect on protein translation. (a) represents the distribution of mutation types that would have different effects on protein synthesis, while (b) represents the distribution of SNVs.

The study was performed on one single female patient who was born to consanguineous parents and presents a clinical history of recurrent typhoid fever from last 4 to 5 years. Following alignment of sequences and variants, exomes were filtered to identify various significant mutations among 25 genes involved in the IL-12/IFN-*γ* axis pathway (Table 1), the distribution of single nucleotide variants (SNVs) region and the effect of these SNVs on protein translation were also determined (Figure 1).

### 3.1. Identification of Putatively Causative IL-12/IFN-*γ* Axis Genes Variants

When exomes were filtered to identify significant mutations, then the analysis was restricted to variants containing the 25 mutations in the IL-12/IFN-*γ* axis, known to be associated with recurrent typhoid fever, eight genes with nonsynonymous, and heterozygous mutations were observed. Identified variants were then analyzed for various predictors of deleterious and pathogenic mutations (Table 2). Deleterious mutations were found in 2 of the 8 variants (25%), IL-23R and ZNFX1 (c. 9G>T, c. 929T>C, and c. 3777G>A), respectively. Moreover, the value for ExAC, CADD, and gnomAD (Genome Aggregation Database) was also calculated as shown in Table 3. The qualitative associations between normal and disease variants of normal and diseases patient are independent on each other as demonstrated by statistical analysis.

## 4. Discussion

Host immune response against intracellular bacteria is mediated via the IL-12/IFN-*γ* axis pathway. IFN-*γ* is the major cytokine involved in the immune response against intracellular bacteria. The primordial function of this cytokine is activation of macrophages, exerting its antibiotic function [11] In this study, whole exome sequence (WES) data of one female patient who had been suffering from recurrent typhoid fever for the last several years were investigated. WES is a recent tool that gives a better understanding of the insight of the organism's genome by finding novel targets that further help in the diagnosis of the patients [22].

The ultimate goal of this study was to identify any pathogenic and deleterious single nucleotide variants in the IL-12/IFN-*γ* axis that might be associated with recurrent intracellular infection particularly caused by *S. typhi*. As limited sufficient data is available regarding the importance of the IL-12/IFN-*γ* pathway in recurrent typhoid infection caused by *S. typhi* in our region, the study attempts to explain the importance of various genes involved IL-12/IFN-*γ* pathway against recurrent typhoid infection. The collective reports of patients having defects in the IL-12/IFN-*γ* axis showed a high occurrence of intracellular infection with a high rate of consanguineous marriages and a low occurrence of HIV, which makes it useful for screening of defects in the IL-12*/*IFN-*γ* axis pathways. Earlier screening of a patient with immune deficiencies will not only improve the quality of life but will also improve the health of the patients suffering from these defects [2]. A single nucleotide mutation (c. 9G>T, c. 929T>C, and c. 3777G>A) was found in IL-23R and ZNFX1, respectively. These single nucleotide variants, were detected via WES as suggested by Meyts et al. [19].

Mendelian susceptibility to mycobacterial disease (MSMD) is a rare disorder that affects individuals upon infection with the weakened strain of *Mycobacterium tuberculosis*. However, the genetic etiology of numerous patients with this disease has remained unidentified. Patients with MSMD are often vulnerable to *Mycobacterium tuberculosis* and *Salmonella spp*. Initially, *Salmonella* infection was reported as a coinfection with *Mycobacterium tuberculosis*, but later on, patients with *S. typhi* with impaired immunity and in the absence of confections with *Mycobacterium tuberculosis* were identified (Dusan et al., 2013; [11, 18]. Results of the current study indicate the importance of the IL-12/IFN-*γ* axis in providing immunity to *S. typhi* and defects in this pathway will be led to the susceptibility to infection for both *S. Typhi* and *M. tuberculosis* [23].

IL-23 is a heterodimeric cytokine released in response to microbial stimulation by dendritic cells. This cytokine acts on the corresponding T cells to induce the production of various cytokines such as IL-17 and IL-22. This pathway is known as the IL-23 axis. Thus, individuals with mutations in either IFN-*γ* or IL-23 or both are susceptible to infection with *Salmonella spp*. *[*10*]*. The results of the current study are consistent with this description since deleterious mutations were found in the IL-23 cytokines. In addition, reported mutation in the p.Q3H and p.L310P of IL23R underlies MSMD corroborating the recent finding of Staels et al. with an R381X mutation in IL23R [7].

In the current study, we reported deleterious mutation of the exon 14 (c. 3777G>A) of the ZNFX1 gene with no deleterious mutation in IFN-*γ*, which shows that the production of normal IFN-*γ* can occur in ZNFX1 deficient lymphocytes. Our results are concurrent with the study conducted by which shows that normal production of IFN-*γ*. Similarly, the single nucleotide mutation in ZNFX1 mutation is responsible for causing intracellular bacterial infection but was without any inborn defect of IFN-*γ* [14].

Characterization of inborn defects of the IL-12/IFN-*γ* axis against recurrent typhoid fever is still to be explored with greater emphasis on patients with recurrent typhoid fever. Furthermore, an investigation is required to characterize the similar inborn single nucleotide mutations in genes to those discussed here (IL-23R and ZNFX1), which caused recurrent typhoid fever. Similar investigations should be conducted in countries where the prevalence of typhoid fever is high and where epidemics caused by *S. typhi* occur.

## 5. Conclusion

The results of the current study investigated the spectrum of mutations associated with deficiency of IL-12/IFN-*γ* axis gene in recurrent typhoid fever patient. Applying whole exome sequencing (WGS) to the patient with recurrent typhoid fever detected variants that are not as important as other genes in the IL-12/IFN-*γ* axis. This study provides a baseline data, therefore, further studies with a large population sizes are needed to examine the functional relevance of IL-12/IFN-*γ* axis genes with recurrent typhoid fever.

## Figures and Tables

**Table 1:** Sequencing summary.

S.no.	Sample	S_2_AH5YFTDSX2_L4
1	Raw reads	45, 423, 136
2	Raw bases (G)	13.6269
3	Clean read	45, 288, 650
4	Clean bases (G)	13.5238
5	Effective rate (%)	99.24
6	Q20 (%)	98.27
7	Q30 (%)	94.92
8	Error rate (%)	0.020
9	GC ration (%)	48.30

**Table 2:** Putatively causative IL-12/IFN-*γ* axis genes variants.

S.no.	Gene	Chrom	Position	REF	ALT	SIFT_ pred	Polyphen2 _HDIV_ pred	Polyphen2_HVAR_ pred	LRT_ pred	MutationTaster_ pred	MutationAssessor_ pred	FATHMM_ pred
1	Interferon gamma receptor 2 (interferon gamma transducer 1)	chr21	34787312	A	G	T	B	B	N	P	N	T
2a	Interleukin 23 receptor	chr1	67633812	G	T	T	B	B	N	P	M	D
2b	Interleukin 23 receptor	chr1	67685387	T	C	T	B	B	N	P	N	T
3	ISG15 ubiquitin-like modifier	chr1	949608	G	A	T	B	B	N	P	L	T
4a	Zinc finger, NFX1-type containing 1	chr20	47865509	G	A	T	B	B	N	P	N	D
4b	Zinc finger, NFX1-type containing 1	chr20	47872377	C	G	T	B	B	N	P	L	T
5	Nuclear factor of kappa light polypeptide gene enhancer in B cells inhibitor-like 1	chr6	31525912	C	T	T	B	B	N	P	—	T
6	Tyrosine kinase 2	chr19	10475649	C	T	T	B	B	N	N	N	T
7	Interleukin 4 receptor	chr16	27374400	A	G	T	B	B	N	P	N	T
8	Interleukin 17 receptor A	Chr22	17589209	C	T	T	B	B	N	P	L	T

B: benign, D: deleterious, L: lethal, N: neutral, M: medium T: tolerant, P: pathogenic/possible damaging.

**Table 3:** Mono allelic and biallelic mutation along found in variants.

Gene	Refseq transcript	Exon	Coding change	Protein change	CADD_raw	CADD_Phred	ExAC_ALL	ExAC_EAS	gnomAD_exome_EAS
Interleukin 23 receptor	NM_144701	exon2	c.G9T	p.Q3H	2.109	16.92	0.5285	0.6498	0.6438
	NM_144701	exon7	c.T929C	p.L310P	-0.55	0.165	0.8776	0.8776	0.9856
Zinc finger, NFX1-type containing 1	NM_021035	exon14	c.G3777A	p.M1259I	0.1752	0.0439	-1.932	0.001	0.0418

## Data Availability

All data pertinent to this manuscript are presented within this article.
